# Nondestructive monitoring of polyphenols and caffeine during green tea processing using Vis‐NIR spectroscopy

**DOI:** 10.1002/fsn3.1861

**Published:** 2020-09-15

**Authors:** Alireza Sanaeifar, Xinyao Huang, Mengyuan Chen, Zhangfeng Zhao, Yifan Ji, Xiaoli Li, Yong He, Yi Zhu, Xi Chen, Xinxin Yu

**Affiliations:** ^1^ College of Biosystems Engineering and Food Science Zhejiang University Hangzhou China; ^2^ College of Mechanical Engineering Zhejiang University of Technology Hangzhou China

**Keywords:** caffeine, green tea, leaf processing, polyphenols, quantification model, visible and near‐infrared (Vis‐NIR) spectroscopy

## Abstract

Increasing consumption of green tea is attributed to the beneficial effects of its constituents, especially polyphenols, on human health, which can be varied during leaf processing. Processing technology has the most important effect on green tea quality. This study investigated the system dynamics of eight catechins, gallic acid, and caffeine in the processing of two varieties of tea, from fresh leaves to finished tea. It was found that complex biochemical changes can occur through hydrolysis under different humidity and heating conditions during the tea processing. This process had a significant effect on catechin composition in the finished tea. The potential application of visible and near‐infrared (Vis‐NIR) spectroscopy for fast monitoring polyphenol and caffeine contents in tea leaves during the processing procedure has been investigated. It was found that a combination of PCA (principal component analysis) and Vis‐NIR spectroscopy can successfully classify the two varieties of tea samples and the five tea processing procedures, while quantitative determination of the constituents was realized by combined regression analysis and Vis‐NIR spectra. Furthermore, successive projections algorithm (SPA) was proposed to extract and optimize spectral variables that reflected the molecular characteristics of the constituents for the development of determination models. Modeling results showed that the models had good predictability and robustness based on the extracted spectral characteristics. The coefficients of determination for all calibration sets and prediction sets were higher than 0.862 and 0.834, respectively, which indicated high capability of Vis‐NIR spectroscopy for the determination of the constituents during the leaf processing. Meanwhile, this analytical method could quickly monitor quality characteristics and provide feedback for real‐time controlling of tea processing machines. Furthermore, the study on complex biochemical changes that occurred during the tea processing would provide a theoretical basis for improving the content of quality components and effective controlling processes.

## Graphical abstract

Green tea polyphenols and caffeine are affected by variety and leaf processing step. Vis‐NIR spectra can distinguish different varieties and processing steps. This approach could quickly detect quality characteristics during leaf processing.

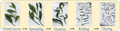

## 1 INTRODUCTION

Tea, as one of the most common alcohol‐free and caffeinated drinks, has a wide market around the world. Green tea production and consumption have considerably grown during the last decade, and this trend is expected to continue due to its highlighted benefits in health and disease prevention worldwide (Ahmed & Stepp, 2013). As the origin of tea, China has a long history of drinking tea and long‐standing tea culture, especially green tea. China as the main tea exporter in the world has still faced with a lack of competitiveness of tea produced in the international market, which is mainly due to the deficiency in product quality and processing methods of domestic tea (Choung, 2010). Several procedures are needed for the manufacturing of green tea including fresh leaves, spreading, fixing, rolling, and drying. The organic compounds formed during processing cause the unique characteristics of green tea, and their changes play an effective role in forming the characteristic green color and aromas (Gao et al., 2017). Traditional green tea processing is done manually by tea makers, but in today's society, with the increasing demand for tea and the development of science and technology, industrial mechanization is expected to become the mainstream approach in the tea production. In traditional processes, the quality of the product is closely related to the experience and technology of the tea maker and also raw tea quality, and there is no unified standard. In order to use modern industrial technology to make tea, it is necessary to have a clear understanding and scientifically grading standards of the main components that affect the quality of tea during the processing operations (Tang et al., 2015). The quality of tea is essential for the development of the tea industry, tea farmers’ income, and the health of tea drinkers. Catechin, as one of the main components, helps to form the color, aroma, and taste of tea, and it is also the main health‐beneficial ingredient in tea (Zuo, Chen, & Deng, 2002). Green tea is an excellent source of catechins, as primary polyphenols, up to 20%–30% of the green tea dry weight. Green tea also contains other polyphenols such as gallic acid, the main tea polyphenolic acid, and certain amounts of alkaloids such as caffeine. These compositions of green tea vary according to its variety, climate, horticultural conditions, and particularly technologies applied during the manufacturing process (Araya‐Farias, Gaudreau, Rozoy, & Bazinet, 2014). The main chemical changes of tea polyphenols during green tea processing are oxidation, hydrolysis, polymerization, and transformation. The content of tea polyphenols is one of the most important indicators affecting the quality of tea, and now, sensory evaluation or chemical methods are used in most of the measurements of tea quality (Zhen, 2002). Due to the interference of human factors and external environment, the result of sensory evaluation is subjective and uncertain. Although the chemical methods usually can accurately detect the composition and content of substances in tea, their steps are time‐consuming, tedious, expensive, and destructive and they are not easy to popularize, so it cannot be applied to the rapid nondestructive detection of tea. Therefore, it is important to establish a simple and reliable analytical approach for the measurement of constituent concentrations in tea leaves for developing high‐quality tea products. The spectral analysis technology of near‐infrared spectroscopy (NIRS) is widely used in the qualitative and quantitative detection of components and contents in various tea production processes because of its unique advantages (Bian et al., 2013).

The near‐infrared spectral region is consistent with the vibrational frequency combination of hydrogen‐containing groups (e.g. O‐H, N‐H, and C‐H) in organic molecules and the absorption region of frequency doubling at all levels. So the characteristic information of hydrogen‐containing groups in organic molecules can be obtained by scanning the near‐infrared spectra of samples (Sandorfy, Buchet, & Lachenal, 2006). The use of near‐infrared spectroscopy to analyze samples is a convenient, fast, efficient, accurate, and low‐cost way; in addition, it does not destroy the sample, consume chemical reagents, and pollute the environment and other characteristics (Chen et al., 2003). It has been widely used in agricultural and nonstaple food, energy and chemical industry, biopharmaceutical, and other fields (Chen, Zhao, Chaitep, & Guo, 2009; Wold et al., 2020; Zareef et al., 2020). Some scholars used visible–near‐infrared spectroscopy combined with chemometrics to quantitatively analyze the tea polyphenol content of Tieguanyin tea (Chen, Zhao, Zhang, & Wang, 2006), the moisture content in tea drying process (El‐Shahawi, Hamza, Bahaffi, Al‐Sibaai, & Abduljabbar, 2012), the crude fiber, moisture, and ash content in tea (Khan & Mukhtar, 2007), and caffeine, total catechins, and four individual catechins in instant green tea (Sun et al., 2020); classify six different commercial tea products (oolong, green, yellow, white, black, and Pu‐erh) (Mishra et al., 2018); and discriminate special‐grade flat green tea (Li, Guo, et al., 2019). Although there are many other related studies, the reports on the detection of tea polyphenols by near‐infrared spectroscopy of the process of green tea processing are few and not detailed enough. Therefore, this paper applies near‐infrared spectroscopy to detect the content of polyphenols according to green tea processing procedures.

It is worth noting that a large amount of spectral data can cause strong redundancy and analysis complexity. Therefore, if we directly deal with the original data, it is likely that the accuracy of the model is not enough to precisely predict the parameters. A suitable algorithm not only reduces data redundancy and removes uninformative and interfering variables but also simplifies model calculations and increases model accuracy (Le Gall, Colquhoun, & Defernez, 2004). In this paper, successive projections algorithm (SPA) is used to select the characteristic wavelengths.

The tea quality characteristics are closely related to the changes in quality components during the processing of green tea. The research on chemical regulation of tea processing quality is one of the most important challenges in the tea industry. Recognizing the changes and key control points may improve the tea quality and optimize the tea processing condition (Zhen, 2002). So, the aims of this study were as follows: (a) to investigate the effects of variety and processing procedure on catechin, gallic acid, and caffeine contents of green tea; (b) to develop a rapid, simple method for determination of polyphenol and caffeine contents based on Vis‐NIR spectroscopy.

## 2 MATERIALS AND METHODS

### 2.1 Sample preparation

**Figure 1:**
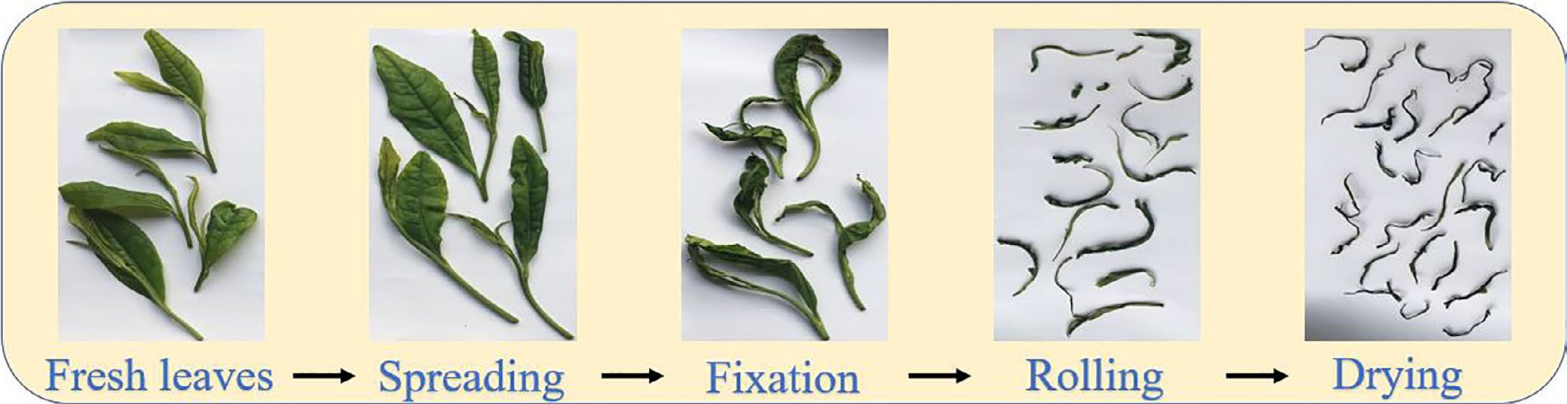
Green tea samples during the processing procedure

Tea leaves of two varieties of Zhongcha108 and Longjing43 were plucked from Fuyang Tea Factory, Hangzhou, Zhejiang Province. Leaves during processing procedure can be divided into five steps: fresh leaves, spreading, fixation, rolling, and drying, as shown in Figure 1. Ten samples containing about 10 g of tea were collected for each step from the two varieties. As a result, a total of 100 tea samples were collected in this study.

### 2.2 Spectra collection

The foliar Vis‐NIR spectra were recorded in the reflectance mode using an XDS near‐infrared spectrometer (FOSS, Denmark). Each spectrum was the average spectrum of 32 scans at a resolution of 2 nm over a range of 400–2,498 nm. Tea leaves (approx. 10 g) were uniformly placed at the sample container, and there is no gap between leaves to avoid affecting the spectrum collection. To obtain the detection data closest to the real tea processing, no additional processing was done to the samples. To increase the signal‐to‐noise ratio, each tea sample was measured 3 times and the average of the three spectra was employed for further analysis. The spectra collection was conducted by ISIscan 1.50 (Infrasoft International LLC, State College, PA, USA). The whole operations were performed at room temperature (about 25°C).

### 2.3 HPLC detection

#### 2.3.1 Chromatographic detection conditions

The contents of constituents were determined by a Shimadzu LC‐2‐AD HPLC system (Shimadzu, Japan) coupled with an ultraviolet–visible (UV–Vis) detector (wavelength range: 190–600 nm). Separations were conducted on a Shim‐pack VP‐ODS‐C18 column (particle size: 5 μm, 250 mm × 4.6 mm) (Shimadzu Co.). Mobile phase A was acetic acid/acetonitrile/distilled water (0.5:3:96.5, v/v/v), and mobile phase B was acetic acid/acetonitrile/distilled water (0.5:30:69.5, v/v/v). Separations were performed with the following solvent gradient procedure: 0‐35min, linear gradient from 20% to 65% B; 35‐45min, isocratic at 65% B. The flow rate was 1.0 ml/min, and the injection volume was 10 μl. The UV detection wavelength was 280 nm, and the column temperature was set at 35°C.

2.3.2. Reference analysis of the chemical constituents.

To quantitatively analyze the constituents of tea samples by HPLC, the standard substances of epigallocatechin (EGC), epicatechin (EC), epigallocatechin gallate (EGCG), gallocatechin gallate (GCG), catechin gallate (CG), epicatechin gallate (ECG), catechin (C), gallocatechin (GC), gallic acid (GA), and caffeine (CAF) with a purity of higher than 98% were purchased from Chengdu Purechem‐Standard Co. LTD, Sichuan, China.

The samples were dried in a freeze‐dryer (FreeZone 6, Labconco Corp., Kansas City, MO, USA) for 24–28 hr. The samples before and after drying were weighed to calculate the moisture content. Then, the samples were milled into powder with a grinder (FW100, Taisite Instrument Co., Ltd.) and sieved through a 40‐mesh sieve. Subsequently, 0.1 g of the sieved tea powder of each sample was weighed into a test tube, 25 ml pure water was added, and the mixture was stirred. The test tube was stored for 20 min in a water bath at 85°C. The supernatant was then filtered out with a 0.22‐μm membrane filter and injected into the HPLC system (He, Zhao, Zhang, Sun, & Li, 2019). The operations were conducted at room temperature and dark environment to limit the decomposition of tea polyphenols.

### 2.4 Data analysis

As the factors affecting the quantitative detection of visible–near‐infrared spectroscopy are complex, the sample set should be divided scientifically before establishing the model to reduce some unnecessary random errors and better evaluate the effect of the model. In this experiment, the sample set was randomly divided into calibration set and prediction set at a ratio of 2:1. All 100 samples were first arranged in ascending order according to chemical content values, and one sample was selected from every three samples consecutively for the prediction set to test the robustness of the model. In addition, this experiment also uses the method of full cross‐validation (leave‐one‐out cross‐validation) to ensure the stability and accuracy of the model. The performances of the developed models were evaluated based on performance criteria including the coefficient of determination for the calibration set ($R_{c}^{2}$), validation set ($R_{v}^{2}$), and prediction set ($R_{p}^{2}$) and the root‐mean‐square error of the calibration set (RMSEC), validation set (RMSEV), and prediction set (RMSEP). Models with high *R*_2_ and low RMSE values were considered to be efficient (Zhou, Li, He, & Jin, 2018).

Vis‐NIR spectroscopy usually consists of thousands of variables, and there are a large number of redundant data within the full spectrum as the input variables. The successive projections algorithm (SPA) is a useful method, which can acquire a small subset of variables with a minimum collinearity (Huang et al., 2019). In this research, it was used for characteristic wavelength selection. Selecting the variable set with the least redundancy from the spectral information can help reduce the number of the variables to the minimum and eliminate collinearity between variables.

To visualize differences between different processing steps and varieties, principal component analysis (PCA) was applied. PCA is one of the most widely used classification procedures to extract the overall characteristics of spectral data, which has robust data compression capability (Magnaghi et al., 2020). PCA is a linear transformation that transforms original variables into a new set of variables, which effectively reduces the dimension while maintaining most of the characteristics of the original data. This method has a good dimension reduction effect by limiting the collinearity between variables (Sanaeifar, Mohtasebi, Ghasemi‐Varnamkhasti, Ahmadi, & Lozano, 2014). To establish an efficient and stable regression model, partial least squares (PLS) algorithm was first used based on the full spectrum. PLS is the most commonly used classical multiple linear regression method in spectral data analysis which projects the high‐dimensional data space into a set of new variables that are orthogonal to each other, namely latent variables (LVs) (Biancolillo, Santoro, Firmani, & Marini, 2020). PLS eliminates the influence of unhelpful noises on regression and makes a model containing a minimum number of variables (Chiesa et al., 2016). Moreover, multiple linear regression (MLR) is a statistical approach, which analyzes the linear relationship between two or more independent variables (X) and one dependent variable (Y) (Li, Jin, Sun, Ye, & Liu, 2019). This method was applied to develop simplified models based on the characteristic wavelengths selected by SPA. The software of MATLAB (2017b) (The MathWorks, Inc.) and Unscrambler 10.3 (CAMO AS, Trondheim, Norway) was used for these analyses.

## 3 RESULTS AND DISCUSSION

### 3.1 Variation of catechin, gallic acid, and caffeine contents of tea samples during green tea processing

**Figure 2:**
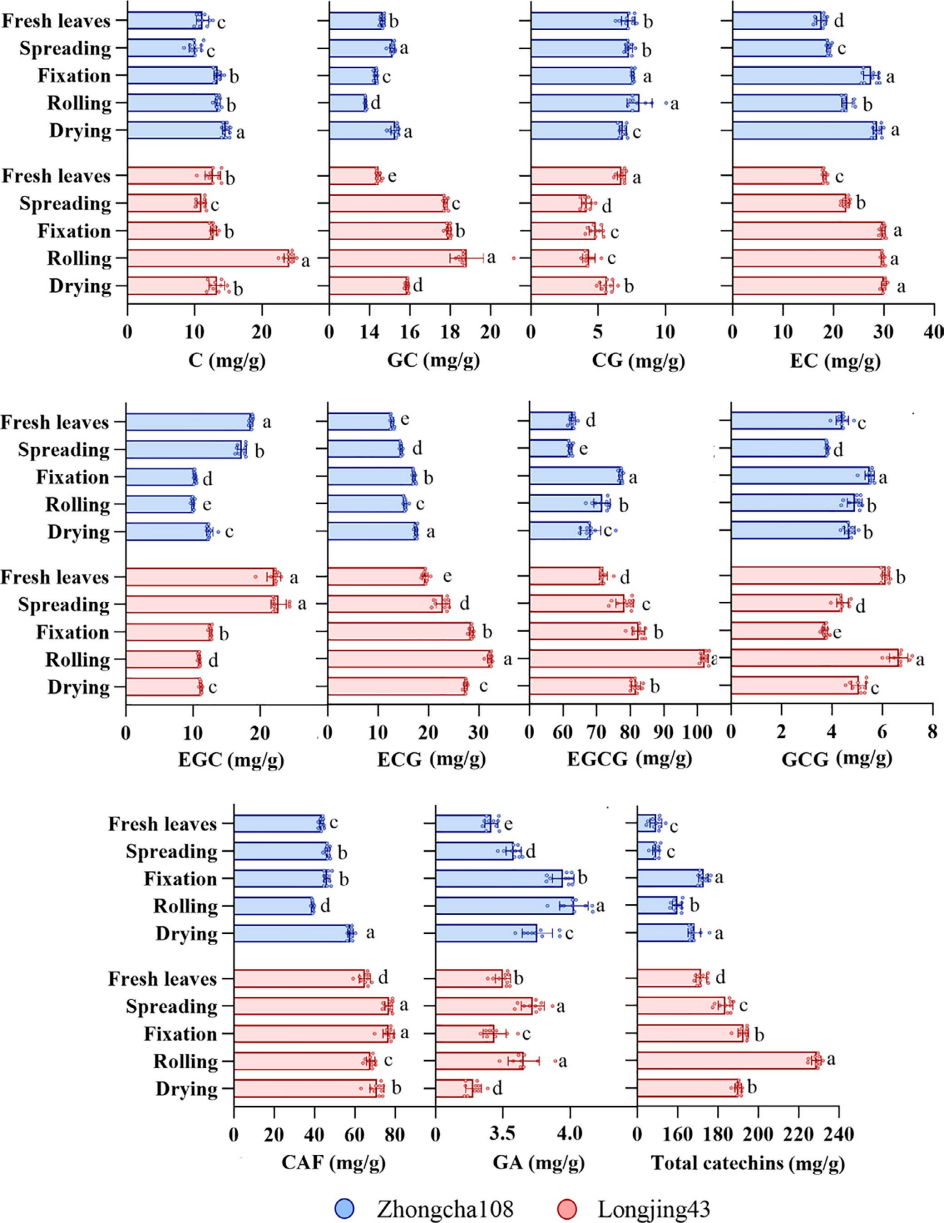
Variation of catechin, gallic acid, and caffeine contents during green tea processing in two varieties

The moisture content of tea samples during the processing procedure in two varieties is shown in Table 1. It can be seen that the water content changes particularly during the processing, and the quality of the tea is formed under the drastic change of water and heat. The contents of catechins, gallic acid, and caffeine in green tea samples were detected as references based on HPLC, and the concentrations for different processing steps and different varieties are presented in Figure 2.

**Table 1:** Average moisture content during green tea processing

	Fresh leaves	Spreading	Fixation	Rolling	Drying
Longjing43	79.12%	69.21%	47.26%	10.09%	3.91%
Zhongcha108	80.92%	69.30%	41.87%	9.58%	4.83%

The enzymes responsible for oxidation are deactivated through the green tea processing, and this procedure is essential in stabilizing and increasing the shelf life of catechins (Ahmed & Stepp, 2013). Leaf spreading is considered as an essential treatment before fixing for processing of high‐quality green tea. Spreading out of green leaves can enhance the formation and accumulation of nongallated catechins (EGC, EC, GC, and C) and change of some physical and chemical properties and loss of moisture in fresh leaves for more stable fixation. The main purpose of fixing is to inactivate enzymes, especially polyphenol oxidase, which prevents the oxidation of tea polyphenols (mainly catechins) and keeps the green color of the leaves. The higher temperature in a long time is needed for fixing tender leaves as they have higher water content and enzyme activities than mature leaves. The quality of green tea is tightly dependent on suitable temperature and time of fixing. It is shown in Figure 2 that the content of ECG, CG, C, EC, and EGCG for two varieties increased in the fixation step; the reason is probably that catechins in fresh tea leaves are extremely unstable in the process of fixation under the conditions of high temperature, high humidity, and oxygen. So, leaves can easily experience a series of changes, such as oxidation, pyrolysis, polymerization, and transformation, which led to an increase in the total catechins (TC) and obvious changes in catechin monomers. Rolling is also an important step in processing green tea. During rolling, leaf cells are destroyed and leaf juices are released and leaf's shape is changed to twist. The degree of pressure, rolling time, rolling technique, and leaf temperature are important technical parameters. For Longjing43 variety, the rolling was the highest among the processing steps in TC, ECG, GC, GCG, C, and EGCG content. For Zhongcha108 variety, the fixation had the highest amount in TC content and the rolling step was the highest one in ECG, CAF, GC, C, and EC content.

**Figure 3:**
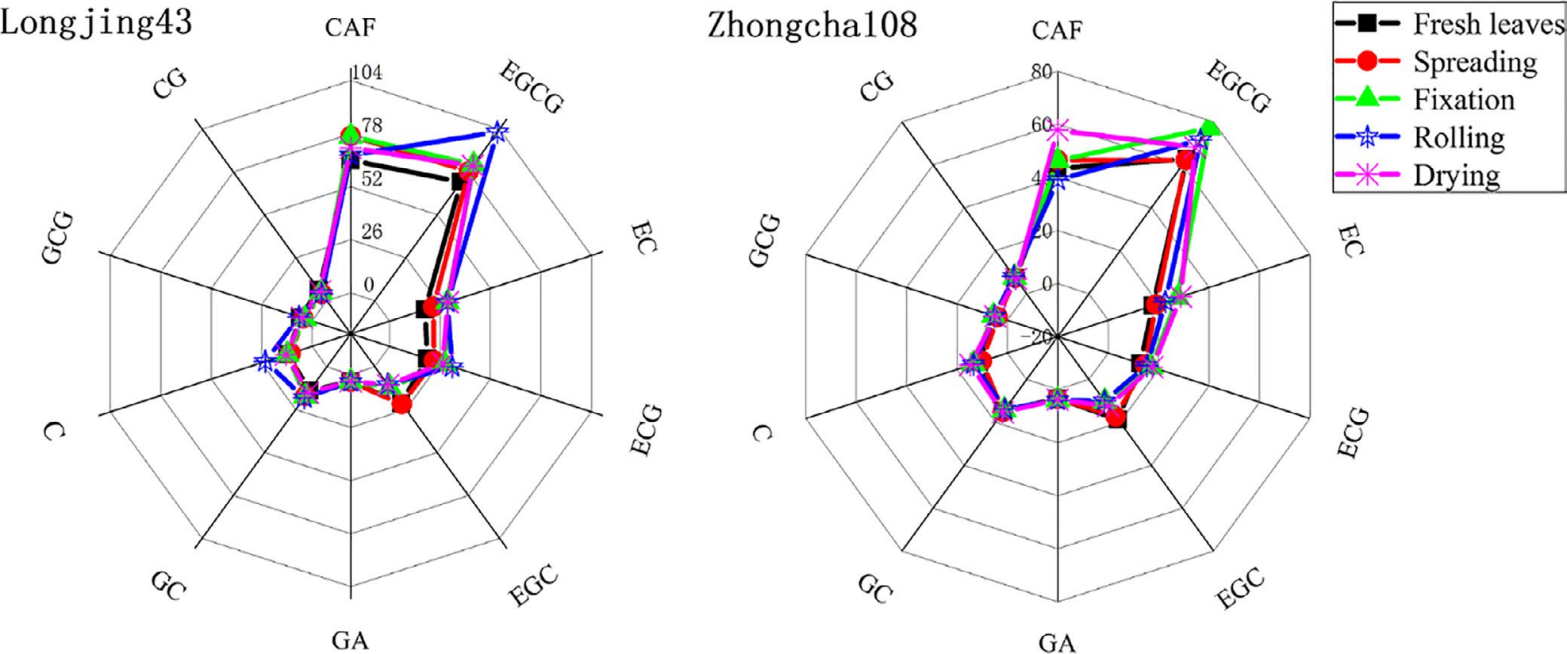
Radar plot in terms of the concentrations of ten constituents from different tea processing steps for two varieties

The hydrolysis of chlorophyll and autooxidation of polyphenols can be due to longer rolling and heavier pressure imposed on fixed leaves. The drying step is usually conducted several times, and this step has the main role to further remove moisture, create the shape, produce aroma compounds in tea, and promote the further transformation of ester catechin to nonester catechin (simple catechin) (Zhen, 2002). There was no significant difference in TC content between fixation and drying steps for Longjing43 variety and fresh leaves and spreading step for Zhongcha108 variety. The contents of GA were also not significantly different in the processing steps. In general, the contents of TC in Longjing43 variety were higher than Zhongcha108 variety, and the maximum and minimum constituents in two varieties were EGCG and GA, respectively (Figure 3). These results are consistent with some previous studies (Han et al., 2016; Lee, Kim, Kim, & Kim, 2014). The present results are consistent with the results of the experiments of Chen, Zhu, Tsang, and Huang, (2001), who found that the content of total catechins is mainly dependent on varieties, brands, and areas of harvest. However, the amount of total catechins was relatively consistent with EGCG and ECG as most abundant, followed by EC and EGC.

In addition, temperature plays an important role in catechin content, as shown in Figure 2; high‐temperature processing led to isomerization or pyrolysis of catechins which caused the TC of two varieties to increase during the processing procedure. Also, Figure 2 shows that the content of simple catechins (C, EC, ECG, and EGCG) increased under the steps of fixation and rolling, which may be caused by that the promotion of the leaching of tea internal substances by the procedure of rolling accelerates the degradation of complex catechins under high temperature and humidity. However, caffeine is less sensitive to heat and is not significantly affected during processing (Ahmed & Stepp, 2013).

**Figure 4:**
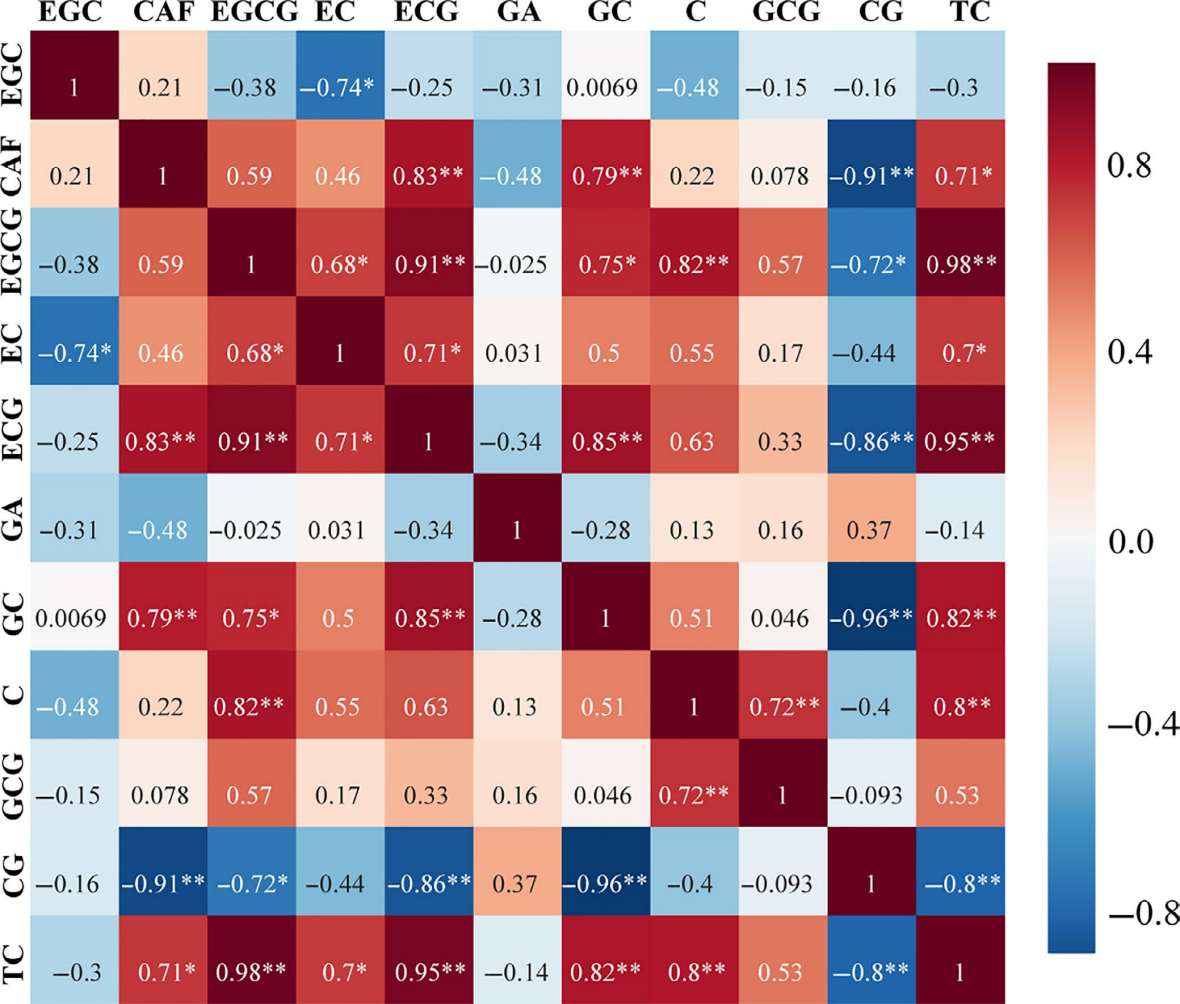
Pearson's correlation coefficients between constituents during green tea processing. *Correlation is significant at the 0.05 level (2‐tailed). **Correlation is significant at the 0.01 level (2‐tailed)

Pearson's correlation coefficients (PCCs) between polyphenol and caffeine contents during green tea processing are shown in Figure 4. It can be used to assess the relationship between constituents and to establish their relative importance in tea processing. Also, it is possible to identify key factors determining the total catechins of green tea and the impact of these factors during the leaf processing. Positive correlations were observed between constituents with PCC ranged from 0.0069 to 0.98, and the highest positive correlation coefficient was obtained between TC and EGCG. According to Figure 4, there were negative correlations with PCC ranged from −0.025 to −0.96, and the highest negative correlation coefficient was found between CG and GC. Results presented in Figure 4 show that TC content had a significant positive correlation with EGCG (*r* = 0.98) and ECG (*r* = 0.95), indicating that an increase in EGCG or ECG corresponds to increased total catechins in the green tea leaves. Also, ECG content demonstrated a strong positive correlation with EGCG (*r* = 0.91) and GC (*r* = 0.91). However, strong negative correlations were found between CG content and the following constituents: CAF (*r* = −0.91), ECG (*r* = −0.86), and GC (*r* = −0.96).

### 3.2 Vis/NIR spectral response of tea samples from tea processing

**Figure 5:**
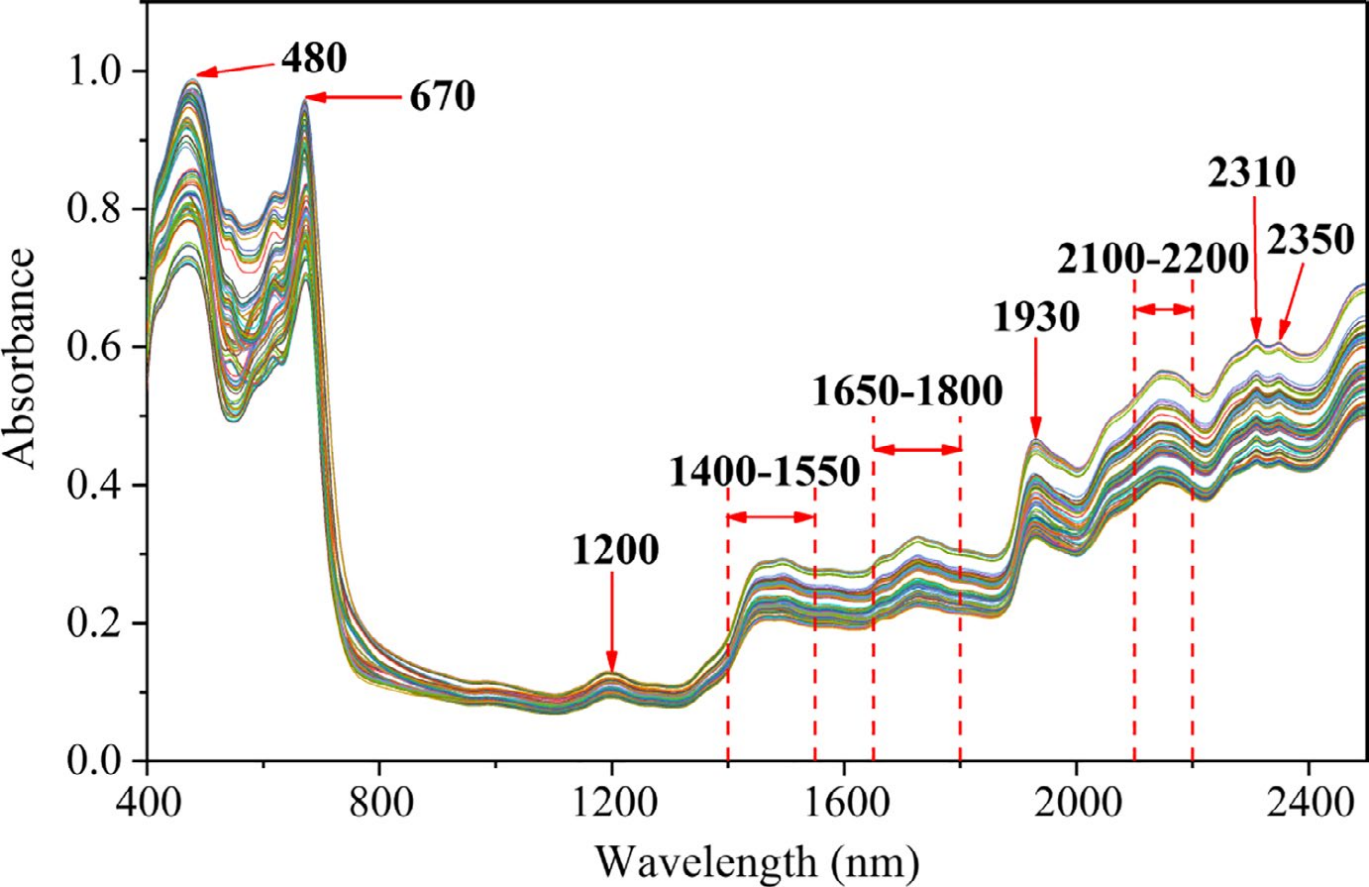
Visible and near‐infrared spectra of tea samples

The raw spectra of tea samples in the wavelength range of 400–2,498 nm are plotted in Figure 5, and the main absorption peaks were marked with wavelength. All tea leaves had a similar pattern in the whole spectral region with diversity in the quantity of absorbance. The absorption peaks at 480 nm and 670 nm were located in the visible region of the spectra (400–800 nm). It proved that the tea leaves mostly absorb light in the visible spectral range (blue‐violet (455–492 nm) and red (622–770 nm)) while green light absorption (500–560 nm) is too low, so the tea leaves look green. Several absorption bands can easily be observed in the NIR region (800–2,498 nm). The small flat absorption peak appeared at 1,200 nm, indicative of the C‐H stretching vibration of ‐CH_2_ or ‐CH_3_. The band at 1400–1,550nm was assigned to the vibration of O‐H, C‐H, and N‐H. The signal in the range of 1650–1,800 nm was attributed to the vibration of C‐H, C=O, and water O‐H. The absorption peak at 1930 nm was obtained from in‐phase vibration of OH stretching mode and structurally arranged water molecules. The band at 2100–2,200 nm was effected by the combination vibration of N‐H bending and C=O stretching. The absorption peaks at 2,310 nm and 2,350 nm were limited so that they were assigned to the C‐H symmetric stretching and C‐H bending (Mark & Workman, 2007; Pu, Ragauskas, Lucia, Naithani, & Jameel, 2008). Consequently, these wavenumbers were linked to several functional groups (CH, CH_2_, CH_3_, C=O, OH, and NH) of polyphenols and caffeine of tea (Xiong et al., 2015).

The content of tea polyphenols and caffeine detected by HPLC method was significantly changed in different varieties and processing steps. However, these differences could hardly be recognized in the spectra in Figure 5. The complex compositions of organic samples may lead to overlapping the absorption bands in several regions of the Vis‐NIR. Therefore, chemometric techniques were needed for spectral analysis. PCA was employed to assess the ability of Vis‐NIR spectroscopy to qualitatively classify samples according to their variety and processing step (Mancini et al., 2020).

**Figure 6:**
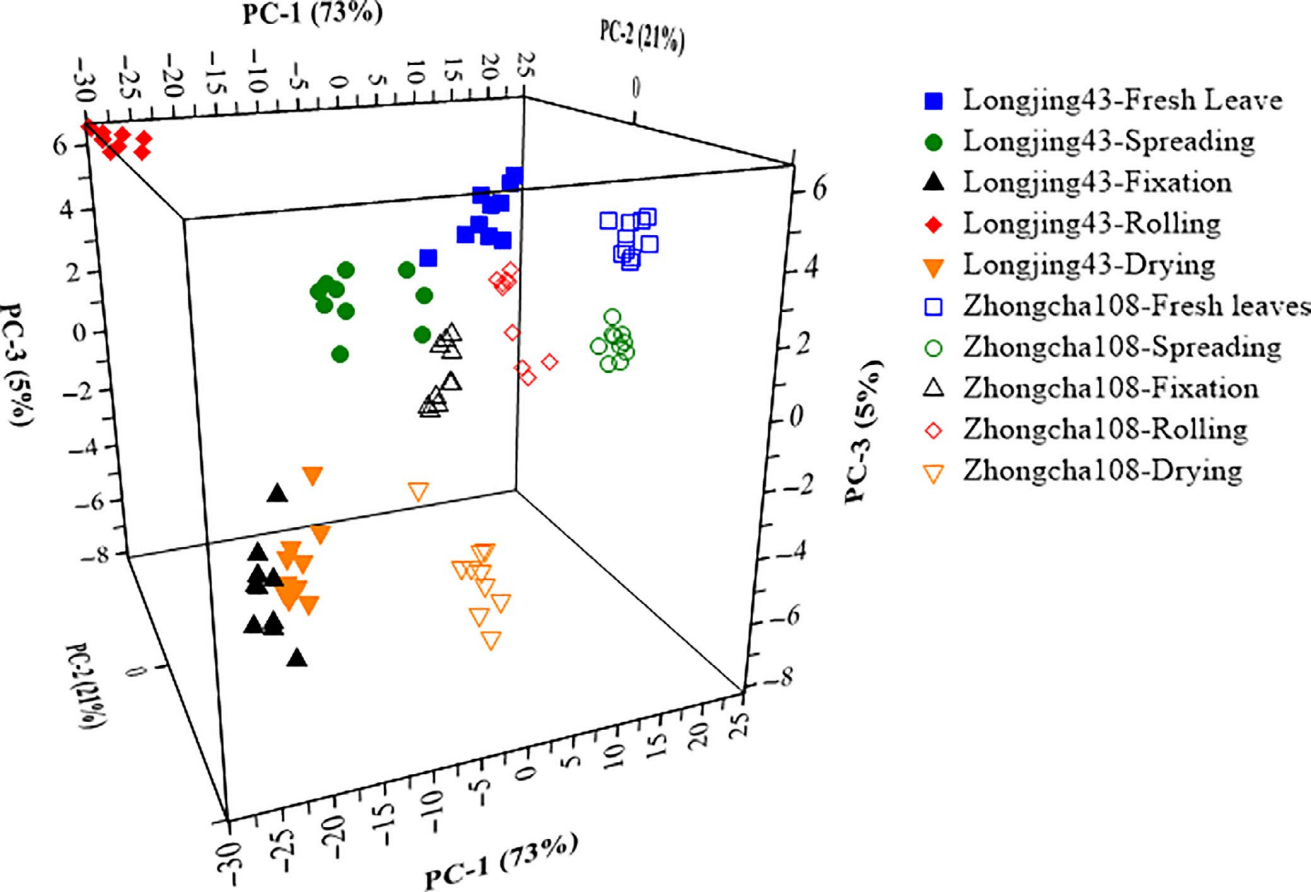
The distribution of all the samples under different tea processing steps in the first three principal component space

Figure 6 shows the result of PCA on the first 3 principal components (PC1, PC2, and PC3) of tea samples that indicated the correlations between the Vis‐NIR spectra and different classes. The results showed that the first three principal components include 73%, 21%, and 5% of data variance in classification based on different varieties and leaf processing steps, which can greatly reduce the dimension of the spectral data. Two varieties of green tea and five processing steps for each variety were separated clearly. It is demonstrated that the differences between samples collected with different leaf processing steps and varieties could be provided by Vis‐NIR spectroscopy combined with PCA.

### 3.3 Spectral detection of constituents during green tea processing

#### 3.3.1 Establishment of regression models based on full spectrum

Establishment of regression models based on full‐spectrum PLS models was developed to build the quantitative relationship between the content of tea polyphenols and caffeine and spectral responses. PLS models were established by taking the data of the whole spectral range as independent variables and each constituent as dependent variable. The modeling results are shown in Table 2. It can be found that $R_{p}^{2}$ values of C and EC were greater than 0.9, and the prediction models of GC, EGC, ECG, EGCG, and CAF showed a great performance with $R_{p}^{2}$ higher than 0.95. It is proved that there were clear quantitative relationships between Vis‐NIR spectra and the polyphenol and caffeine contents, which can be used for nondestructive and rapid detection of the constituents in tea during processing.

**Table 2:** Results of PLS models based on full spectrum

Constituent	$R_{c}^{2}$	RMSEC	$R_{v}^{2}$	RMSEV	$R_{p}^{2}$	RMSEP
C	0.959	0.764	0.938	0.952	0.931	1.002
GC	0.977	0.241	0.971	0.277	0.970	0.278
CG	0.916	0.391	0.889	0.457	0.892	0.487
EC	0.924	1.366	0.914	1.476	0.941	1.182
EGC	0.975	0.734	0.965	0.896	0.981	0.658
ECG	0.988	0.689	0.981	0.880	0.989	0.664
EGCG	0.967	2.012	0.950	2.524	0.964	2.143
GCG	0.946	0.217	0.909	0.286	0.885	0.308
CAF	0.956	2.890	0.936	3.537	0.959	2.757
GA	0.907	0.075	0.849	0.097	0.837	0.094

#### 3.3.2 Establishment of regression models based on characteristic wavelengths

**Figure 7:**
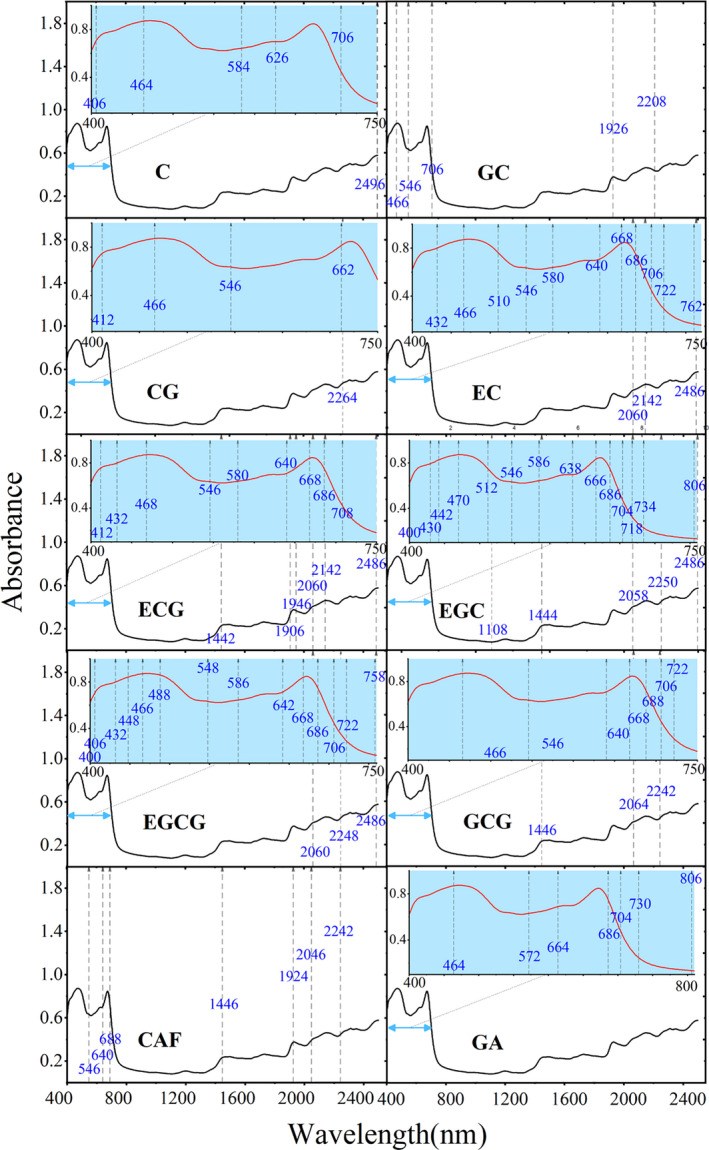
Distributions of the characteristic wavelengths selected by SPA for ten constituents in tea

The reflectance in the visible and NIR is mainly dominated by the plant pigments, compounds, leaf internal structure, leaf anatomy, and the characteristics of the epidermal surface. The vibrational and rotational properties of the molecules present are also controlled with the positions of the absorption features (Croft & Chen, 2017). To efficiently extract spectral information closely related to tea constituents in a large quantity of spectral data, the SPA was used for the selection of characteristic wavelengths. The characteristic wavelengths extracted based on the SPA and their corresponding molecular vibration information are listed in Table 3, and the distributions of the characteristic wavelengths are shown in Figure 7. The characteristic wavelengths extracted by SPA include 6, 5, 5, 14, 15, 19, 18, 10, 7, and 7 wavelengths as the most useful variables for C, GC, CG, EC, ECG, EGC, EGCG, GCG, CAF, and GA, respectively.

**Table 3:** Different functional groups of the characteristic wavelengths for ten constituents

Constituent	Characteristic wavelength (nm)	Functional group	Ref
C	2,496	CH_2_	Lee, Hwang, Lee, and Choung (2014)
GC	1,926	O‐H stretching first overtone	Lee, Hwang, et al. (2014)
	2,208	C=H stretch	Bian et al. (2013)
CG	2,264	O‐H stretching plus C‐H stretching	Choung (2010)
EC	2,060	N‐H asymmetric stretching	Lee, Hwang, et al. (2014)
	2,142	C‐H stretching plus C=C stretching	Lee, Hwang, et al. (2014)
	2,486	CH_2_	Lee, Hwang, et al. (2014)
ECG	1,442	C‐H stretching and C‐H deformation	Bian et al. (2013)
	1,906	O‐H stretching first overtone	Lee, Hwang, et al. (2014)
	1,946	O‐H stretching and HOH transformation	Mark and Workman, (2007)
	2,060	N‐H asymmetric stretching	Lee, Hwang, et al. (2014)
	2,142	C‐H stretching plus C=C stretching	Lee, Hwang, et al. (2014)
	2,486	CH_2_	Lee, Hwang, et al. (2014)
EGC	806	C‐H third overtone	Osborne (1986)
	1,108	C‐H stretching second overtone	Bian et al. (2013)
	1,444	C‐H stretching and C‐H deformation	Bian et al. (2013)
	2,058	N‐H asymmetric stretching	Lee, Hwang, et al. (2014)
	2,250	N‐H stretching and NH_3_ deformation	Huang et al. (2020)
	2,486	CH_2_	Lee, Hwang, et al. (2014)
EGCG	2,060	N‐H asymmetric stretching	Lee, Hwang, et al. (2014)
	2,248	N‐H stretching and NH_3_ deformation	Huang et al. (2020)
	2,486	CH_2_	Lee, Hwang, et al. (2014)
GCG	1,446	C‐H stretching and C‐H deformation	Bian et al. (2013)
	2,064	N‐H asymmetric stretching	Lee, Hwang, et al. (2014)
	2,242	N‐H stretching and NH_3_ deformation	Huang et al. (2020)
CAF	1,446	C‐H stretching and C‐H deformation	Bian et al. (2013)
	1,924	O‐H stretching first overtone	Lee, Hwang, et al. (2014)
	2,046	N‐H asymmetric stretching	Lee, Hwang, et al. (2014)
	2,242	N‐H stretching and NH_3_ deformation	Huang et al. (2020)
GA	806	C‐H third overtone	Osborne (1986)

**Figure 8:**
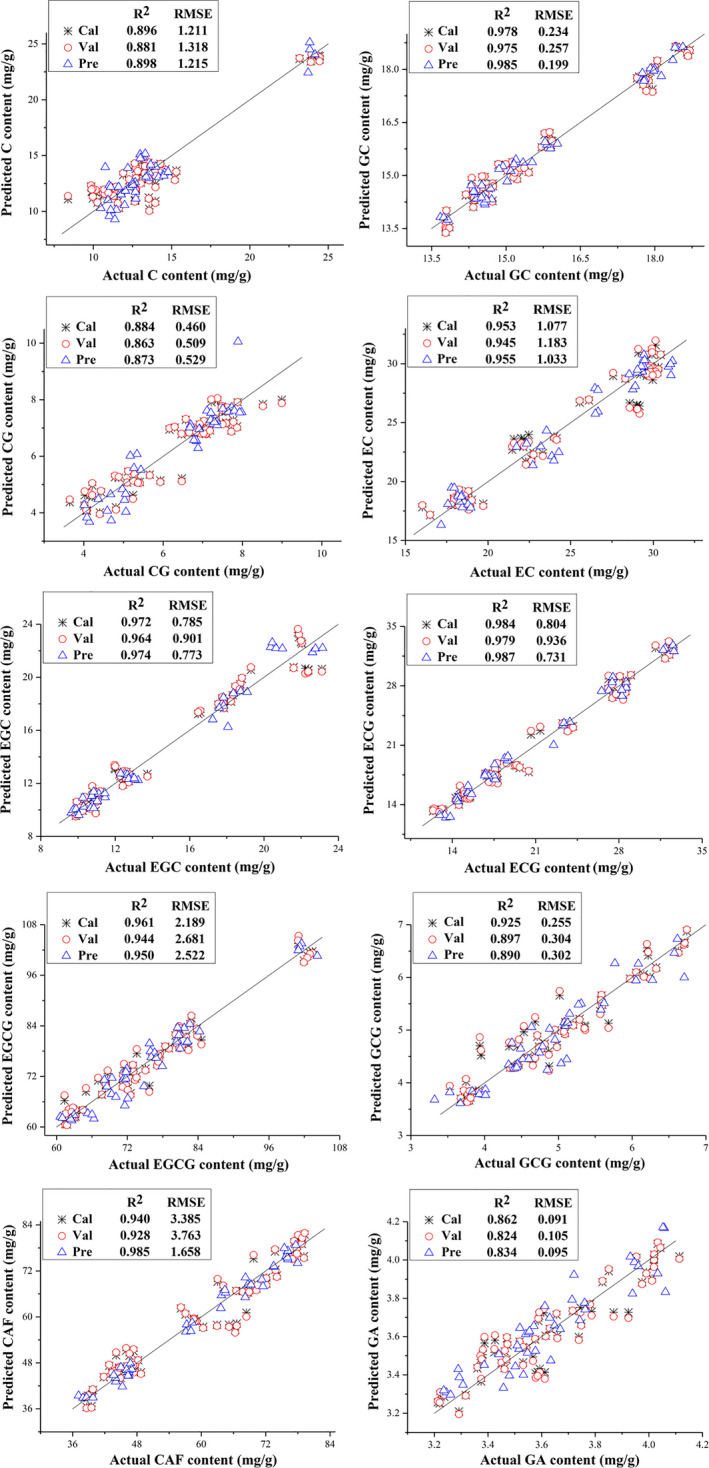
Measured versus predicted contents of ten constituents in tea by MLR models based on the characteristic wavelengths

MLR based on the spectral variable selection of SPA was applied to eliminate collinearity among different wavelengths and simplify the models. The comparison of the performances of MLR models for the polyphenols and caffeine contents is shown in Table 4. Figure 8 shows the correlations between the measured and predicted ten constituents in tea for MLR models, and the scatter distribution for all constituents is concentrated around the diagonals. As shown in Table 3, PLS regression models based on full spectrum had predictive abilities with $R_{p}^{2}$ of greater than 0.837, which means that the accuracy of these models was satisfying. However, all of these models were based on the full wavelength range from 400 to 2,498 nm. A large amount of spectral data leads to produce problems such as large computation, serious collinearity, too much redundant information, and complicated model (Kong et al., 2018). MLR model based on the extracted characteristic wavelengths not only reduces the number of wavelengths but also improves performances with increased $R_{p}^{2}$ values to 0.985, 0.955, 0.890, and 0.985 for GC, EC, GCG, and CAF, respectively. For CG, EGC, ECG, EGCG, and GA, MLR models based on the characteristic wavelengths achieved comparable performance with PLS models based on full spectrum, while the number of variables was significantly reduced. It can be indicated that SPA not only greatly simplified the models with effectively removing the redundant information and successfully extracting the effective wavelengths but also advanced the stability and performance of the detection. Y. Huang et al. (2020) used Vis‐NIR spectroscopy combined with chemometrics as a rapid, simple, and nondestructive approach for the prediction of four main catechin and caffeine contents of tea leaves in three varieties and six leaf positions and built simple detection models for these constituents. MLR models based on characteristic wavelengths had also good prediction performance with $R_{p}^{2}$ greater than 0.893. Furthermore, considerable reduction in the number of input variables helped to establish determinant models with higher modeling speed, which provides the potentiality of real‐time quantitative detection of constituents during green tea processing.

**Table 4:** The performance of MLR models based on the characteristic wavelengths

Constituent	$R_{c}^{2}$	RMSEC	$R_{v}^{2}$	RMSEV	$R_{p}^{2}$	RMSEP
C	0.896	1.211	0.881	1.318	0.898	1.215
GC	0.978	0.234	0.975	0.257	0.985	0.199
CG	0.884	0.460	0.863	0.509	0.873	0.529
EC	0.953	1.077	0.945	1.183	0.955	1.033
EGC	0.972	0.785	0.964	0.901	0.974	0.773
ECG	0.984	0.804	0.979	0.936	0.987	0.731
EGCG	0.961	2.189	0.944	2.681	0.950	2.522
GCG	0.925	0.255	0.897	0.304	0.890	0.302
CAF	0.940	3.385	0.928	3.763	0.985	1.658
GA	0.862	0.091	0.824	0.105	0.834	0.095

## 4 CONCLUSIONS

The results of this research indicate that a combination of Vis‐NIR spectroscopy and chemometrics could determine the polyphenol and caffeine contents in tea leaves from different varieties and processing steps. The contents of catechins and caffeine in green tea are significantly influenced by variety and processing step, and the TC increased during the processing procedure. The differences of spectra in different varieties and leaf processing steps could be presented by PCA, which created a base for the quantitative determination of the content of tea polyphenols and caffeine by Vis‐NIR spectroscopy. Furthermore, this research used the characteristic wavelengths employing the SPA to establish linear models between spectral information and the content of constituents. The results demonstrated that SPA combined with MLR was acceptable as a rapid, simple, and accurate detection method, as *R* ^2^ in all calibration sets and prediction sets was higher than 0.862 and 0.834, respectively. Finally, Vis‐NIR spectroscopy has the potential of becoming a rapid and reliable approach to predict ten major constituents in tea leaves during the processing procedure. Thus, this analytical method has a great potential to quickly track quality characteristics in tea leaves, compared to the traditional methods. The method proposed in this paper could be applied in each step of automatic tea processing production lines to ensure high‐quality processing of tea leaves.

## CONFLICT OF INTEREST

The authors declare no conflict of interest.
